# Interleukin-33 regulates the functional state of microglia

**DOI:** 10.3389/fncel.2022.1012968

**Published:** 2022-11-10

**Authors:** Tianqing Xiong, Xingyi Wang, Yiwen Zha, Yingge Wang

**Affiliations:** ^1^Medical College, Institute of Translational Medicine, Yangzhou University, Yangzhou, China; ^2^Jiangsu Key Laboratory of Experimental & Translational Non-coding RNA Research, Yangzhou, China; ^3^Department of Neurology, Affiliated Hospital of Yangzhou University, Yangzhou, China

**Keywords:** microglia, interleukin-33, phagocytosis, migration, inflammatory response

## Abstract

Microglia, the most prominent resident immune cells, exhibit multiple functional states beyond their immunomodulatory roles. Non-immune functions such as synaptic reorganization, removal of cellular debris, and deposition of abnormal substances are mediated by phagocytosis of normal or enhanced microglia. Activation or migration of microglia occurs when environmental cues are altered. In response to pathological factors, microglia change into various phenotypes, preventing or exacerbating tissue damage. Interleukin-33 (IL-33) is an important cytokine that regulates innate immunity, and microglia are thought to be its target cells. Here, we outline the role of IL-33 in the expression of microglial functions such as phagocytosis, migration, activation, and inflammatory responses. We focus on microglial properties and diverse functional states in health and disease, including the different effects of IL-33 perturbation on microglia *in vivo* and *in vitro*. We also highlight several well-established mechanisms of microglial function mediated by IL-33, which may be initiators and regulators of microglial function and require elucidation and expansion of the underlying mechanisms.

## Introduction

Since microglia were first described in 1919, a high diversity of microglial morphology and function in different environments has been revealed (Prinz et al., 2019). Microglia exhibit an amoeboid morphology and show active phagocytic capacity along with effective proliferation during development (Bennett et al., 2016; Matcovitch-Natan et al., 2016). In the adult brain, microglia tend to be in a homeostatic state characterized by an elevated morphology and continuous immune surveillance (Kettenmann et al., 2013; Madry et al., 2018). In response to central nervous system (CNS) disease, stimulated microglia undergo various structural transformations, cell proliferation, injury-induced migration, substantial mediator production, and enhanced phagocytosis, depending on the given pathology (Tay et al., 2017; Prinz et al., 2019; Borst et al., 2021).

Interleukin-33, a nuclear cytokine of the IL-1 family, is supposed to be a dual function protein (Liew et al., 2016; Cayrol and Girard, 2018). The N-terminal nuclear domain of IL-33 binds to the acidic pocket formed by histone H2A-H2B and promotes chromatin condensation which may represses transcriptional activity (Carriere et al., 2007; Roussel et al., 2008). Importantly, disrupting the nuclear localization of IL-33 results in lethal inflammation triggered by release of IL-33, indicates an intracellular role in sequestration of IL-33 cytokine activity (Bessa et al., 2014). The cytokine activity of IL-33 is dependent of a receptor complex composed of membrane ST2 (ST2L) and IL-1 receptor accessory proteins (Liew et al., 2016). Actually, soluble ST2 (sST2), another isoform of ST2, is the decoy receptor of IL-33 thus can limit extracellular cytokine activity of IL-33 by competing against ST2L (Cayrol and Girard, 2018; Sun et al., 2021).

Despite the fact that IL-33 is highly expressed in the CNS and expression patterns of IL –33 and ST2 have been studied extensively (Schmitz et al., 2005; Gadani et al., 2015; Allan et al., 2016; Liew et al., 2016; Cayrol and Girard, 2018), it is still challenging to provide a definitive answer as to when and where IL-33 and ST2 are expressed in the CNS (Fairlie-Clarke et al., 2018; Sun et al., 2021). One possible reason is that the IL-33/ST2 axis continues from development to adulthood in various CNS regions where different biological processes (Gadani et al., 2015; Cayrol and Girard, 2018; Fairlie-Clarke et al., 2018). Although the distribution of IL-33/ST2 displays temporal and spatial heterogeneity, one of the most investigated target cells of IL-33 in the CNS is microglia, which simultaneously expresses high levels of ST2 in several CNS regions, including thalamus, hippocampus, and the spinal cord (Gadani et al., 2015; Fairlie-Clarke et al., 2018; Nguyen et al., 2020).

Thanks to the benefits of single-cell technology, our knowledge of the sophisticated functional state of microglia as reflected by transcriptional filing has been deeply extended (Keren-Shaul et al., 2017; Hammond et al., 2019; Li et al., 2019; Masuda et al., 2019). What remains unclear, however, is how microglia are uniquely induced to reprogram the transcriptome. Given the critical role of IL-33 in the regulation of innate immunity and the expression pattern of ST2 in the CNS, IL-33 is widely considered to be a prominent adaptor to the functional states of microglia in physiology and pathology.

## Interleukin-33 promotes phagocytosis of microglia

Proper synapse elimination and formation *via* phagocytosis of microglia are essential for functional neural circuitry in normal brain development (Paolicelli et al., 2011; Schafer et al., 2012; Parkhurst et al., 2013; Miyamoto et al., 2016). Although the critical role of microglia in maintaining synaptic homeostasis is gradually becoming more evident, the underlying regulators and specific mechanisms are still poorly understood. So far, IL-33 has been shown to be a key regulatory cytokine for microglia to exhibit adaptive phagocytic functions in neurodevelopment and experience-dependent synaptic plasticity (Vainchtein et al., 2018; Nguyen et al., 2020; He et al., 2022).

The marked expression of ST2 in microglia, as well as the reduction of NF-κB signaling molecules in microglia isolated from IL-33-/- mice, indicate that microglia are affected by or targeted by IL-33 in the healthy CNS (Vainchtein et al., 2018; Nguyen et al., 2020). Notably, different developmental stages and brain regions may have opposite consequences, both related to the phagocytosis of microglia. Direct interactions between microglial processes and dendritic spines induced by astrocyte-derived IL-33 during early thalamogenesis result in enhanced synaptic engagement (Vainchtein et al., 2018). In contrast, the experience-dependent release of IL-33 from adult hippocampal neurons causes microglial remodeling of the extracellular matrix (ECM) and enhances dendritic spine formation and synaptic plasticity (Nguyen et al., 2020). It has been shown that IL-33 promotes phagocytosis of microglia during development, but the mechanism needs to be further investigated. Recently, a correlation between cellular metabolism and microglial phagocytic function has been demonstrated in the developing brain. The IL-33-dependent cluster of microglia in early neurodevelopment was found to have both high mitochondrial activity and phagocytosis-promoting function. Furthermore, treatment with metabolic inhibitors abolished the increase in phagocytic capacity of microglia induced by IL-33, suggesting that mitochondrial activity mechanically regulated by IL-33/ST2/AKT signaling is required for microglia to exhibit phagocytic function during normal development (He et al., 2022). Consistent with the enriched expression of IL-33 in the thalamus and spinal cord of postnatal day 9 (P9) mice, time trajectory proteomic analysis of microglia from P9 to P28 has also shown active phagocytosis during early postnatal development (Vainchtein et al., 2018; He et al., 2022). This phenomenon indicates that IL-33 may enhance phagocytosis of microglia and regulate the neuronal activity, primarily in early development. More convincing evidence, such as perturbations of IL-33 at different time points or complete gene knockout, is needed to support this possibility; IL-33’s critical role in microglial phagocytosis makes it a promising target for neurodevelopmental disorders. However, IL-33 needs further investigation because it may play diverse roles in various brain regions and may cause cellular changes separate from mitochondrial function.

The accumulation of Aβ peptides, one of the typical pathologies of Alzheimer’s disease (AD), leads to chronic neuroinflammation and impaired microglial function, including phagocytosis (Heppner et al., 2015). Several cytokines have been recognized as modulators of Aβ clearance, partially due to phagocytic activity (Vom Berg et al., 2012; Heneka et al., 2013; Chakrabarty et al., 2015; Guillot-Sestier et al., 2015). Among these cytokines, IL-33 significantly promotes Aβ uptake by resident microglia, although it partially alleviates amyloid plaques by increasing Aβ-degrading enzyme expression (Fu et al., 2016).

Following the identification of the requirement for ST2 receptor in microglia during Aβ phagocytosis (Fu et al., 2016), a molecular mechanism for microglial state transitions stimulated by IL-33 has recently been reported that IL-33 induces transcriptome reprogramming characterized by the restoration of homeostatic signature genes and increased expression of major histocompatibility complex class II (MHC-II) genes in a disease-associated microglia (DAM) subpopulation. Furthermore, we found that the expression of MHC-II involved in phagocytosis *via* antigen presentation is upregulated by IL-33-dependent chromatin accessibility remodeling. Enhanced binding affinity between the IL-33-regulated regulatory region of the MHC-II gene and the transcription factor PU.1, which is involved in antigen presentation, ultimately induces augmented MHC-II-expressing microglia and alleviates Aβ deposition (Lau et al., 2020). Indeed, lentivirus-induced IL-33 gain-of-function increased phagocytosis by microglia of the perisynaptic ECM in the aged hippocampus, restoring fewer spine head filopodia numbers found in the dentate gyrus of old mice compared with young adult mice (Nguyen et al., 2020).

**FIGURE 1:**
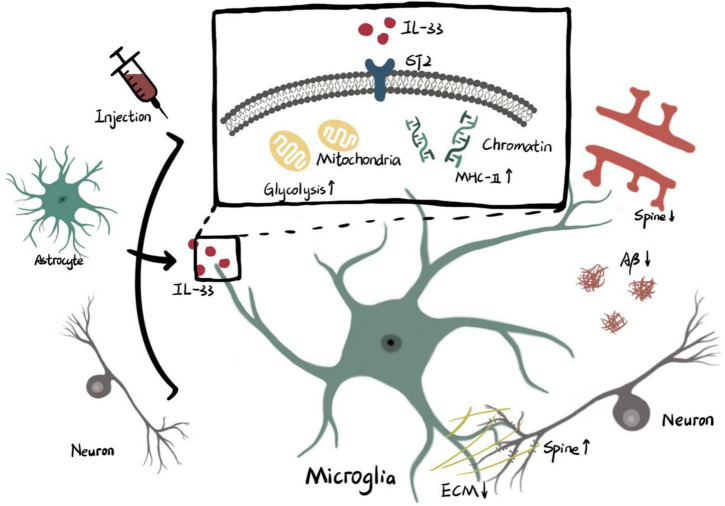
Microglial phagocytosis activity is regulated by IL-33/ST2.

Given the critical role of IL-33 in phagocytosis of microglia during neurodevelopment and AD, and the different mechanisms by which IL-33 is involved (see Figure 1), it is expected that IL-33 will have widely unknown effects on phagocytosis of microglia in health and disease.

Interleukin-33 derived from cellular source or exogenous injection functions on ST2 receptor of microglia. The intracellular effects of IL-33/ST2 axis on microglia include higher level of glycolysis in mitochondria and remodeling of chromatin accessibility which promotes transcription of MHC-II. Enhanced phagocytosis of microglia triggered by metabolic or genetic mechanisms is involved with pruning of spine, clearance of ECM and elimination of Aβ (ECM: extracellular matrix. Aβ: β amyloid).

## Controversy regarding the role of interleukin-33 in microglial activation and migration

In response to environmental challenges or disease, microglia undergo morphological transformations and region-specific accumulation that may result from microglial proliferation or migration (Colonna and Butovsky, 2017; Wolf et al., 2017).

In typical spinal cord sections, knockout of IL-33 (IL-33–/–) induces increased expression of Iba-1 (a classical marker of microglia) (Xiao et al., 2018). Interestingly, another study observed no difference in the number of Iba-1 positive cells in the cortex of IL-33–/– adult mice (Cao et al., 2018). While in the healthy dentate gyrus of the hippocampus, neuronal conditional knockout of IL-33 maintains comparable microglial density when compared with control mice (Nguyen et al., 2020), recombinant IL-33 supplementation leading to elevated microglial cells in the healthy hippocampus (Reverchon et al., 2020). The different results above are probably due to non-specific perturbation of IL-33 (Nguyen et al., 2020; Reverchon et al., 2020) or to the heterogeneous parts of the CNS that were definitively tested (Cao et al., 2018; Xiao et al., 2018).

Permanent deletion of ST2 in microglia impairs the coverage of microglial processes, indicating that IL-33/ST2 signaling induces changes in microglial morphology under conditions in which microglial mass is unchanged (Nguyen et al., 2020). It has also been observed that IL-33 administration increases hippocampal hyper lamellar microglia (Reverchon et al., 2020). The above findings support that the IL-33/ST2 pathway positively influences microglial surveillance.

Independent of uplifted microglial processes, IL-33 induces a tendency for microglia to migrate toward Aβ plaques (Fu et al., 2016). However, the molecular mechanism of microglial recruitment stimulated by IL-33 is still unclear. Interestingly, IL-33 induces the release of chemokines from mixed glial cultures in which microglia are excluded from IL-33 responders, indicating that IL-33 acts indirectly on microglial/macrophage recruitment (Kempuraj et al., 2013; Gadani et al., 2015). Furthermore, glioma cells rich in IL-33 have high CCL7 expression levels and thus promote microglial infiltration. Inexplicably, genetic knockdown of IL-33 results in elevated CCL7 in glioma cells and further promotes microglial migration (Fang et al., 2014). This phenomenon seems to indicate that IL-33 negatively regulates microglial migration in gliomas, explaining the involvement of IL-33 in active glioma growth and invasion. On the other hand, another xenograft glioma study found that glioma-secreted IL-33 with its nuclear domain deleted was sufficient for microglial recruitment (De Boeck et al., 2020). It is quite possible that IL-33 promotes microglial migration in a chemokine-dependent manner, as there is a lack of evidence pointing to a direct role for IL-33 in microglial migration.

Despite the rapid increase in IL-33 expression after transient ischemic stroke and subsequent activation of microglia (Yang et al., 2017), exogenous IL-33 administration does not induce significant microgliosis (Korhonen et al., 2015; Zhang et al., 2018). In contrast, in a model of sciatica resulting from sciatic nerve injury, IL-33 treatment induces robustly activated microglia that are inhibited by ST2–/– (Zarpelon et al., 2016). Additionally, genetic deletion of IL-33 in neuroinflammation induced by lipopolysaccharide (LPS) reduces microglial activation (Cao et al., 2018). Furthermore, the number or activation state of microglia regulated by ST2–/– or IL-33–/– leads in opposite directions in traumatic brain injury and experimental autoimmune encephalomyelitis (EAE), respectively (Wicher et al., 2017; Xiao et al., 2018). Notably, proliferation, often accompanied by marked activation of microglia, has been tested under completely different diseases, which is consistent with the dual function of IL-33 in neuroinflammatory conditions.

In several studies, the relationship between IL-33 and microglial proliferation/migration is unclear. In a model of hypothalamic myelin disruption induced by a high-fat diet, elevated expression of both IL-33 and microglial markers is observed (Huang et al., 2019). WIN 55,212-2 (an agonist for cannabinoid receptors) both promotes IL-33 production and inhibits microglial activation induced by carbon monoxide poisoning (Du et al., 2020). Electroacupuncture combined with induced pluripotent stem cell-derived small extracellular vesicles induces neuroprotection in ischemic stroke through downregulation of the IL-33/ST2 axis and reduction of microglial activation (Deng et al., 2022). These findings indicate a potential role for IL-33 in the stimulation of microglia, but the exact causal role remains unresolved.

Interleukin-33 was suggested to be a fundamental and dynamic regulator of microglial activation and migration upon various stimuli and injuries to the CNS (see Table 1). Given the complex effects of IL-33 on microglial structure and function, it is essential to understand how IL-33/ST2 signaling affects microglia. To investigate the precise effects of IL-33/ST2 signaling on microglial morphology and activation state, it is necessary to temporally manipulate IL-33/ST2 signaling under a variety of conditions.

**TABLE 1:** Controversial roles of IL-33 in microglial activation or proliferation.

Physical and pathological states	Regions	Way of perturbation	Applied techniques	References
Health	Spinal cord	IL-33 KO	Immunofluorescence (Iba-1) ↑	Xiao et al., 2018
	Dentate gyrus	Neuronal IL-33 cKO	Immunofluorescence (Iba-1) −	Nguyen et al., 2020
	Hippocampus	Exogenous rmIL-33	Flow cytometry (CD45lowCD11b +) ↑	Reverchon et al., 2020
	Cortex	IL-33 KO	Immunofluorescence (Iba-1) −	Cao et al., 2018
Ischemic stroke	Peri-ischemic area	Exogenous rmIL-33	Immunofluorescence (Iba-1) −	Korhonen et al., 2015
	Peri-ischemic area	Exogenous rmIL-33	Flow cytometry (CD45lowCD11b +) −	Zhang et al., 2018
Sciatica	Sciatic nerve	Exogenous rmIL-33	Immunofluorescence (Iba-1) ↑	Zarpelon et al., 2016
LPS induced neuroinflammation	Cortex	IL-33 KO	Immunofluorescence (Iba-1) ↓	Cao et al., 2018
Traumatic brain injury	Cortex	ST2 KO	Immunofluorescence (Mac2) ↓	Wicher et al., 2017
Experimental autoimmune encephalomyelitis	Spinal cord	IL-33 KO	Flow cytometry (CD45lowCD11b +) ↑	Xiao et al., 2018

## Interleukin-33 regulates microglial inflammatory responses

Microglia respond to CNS disease is by secreting pro- or anti-inflammatory cytokines (Prinz et al., 2019). This double-edged sword-like effect of microglia is thought to derive from different phenotypic/polar states, mainly classified as M1 and M2 phenotypes. Thus, microglial polarization has been recognized as a promising target for immunomodulatory therapy in neuroinflammation (Hu et al., 2015).

Interleukin-33 treatment is sufficient to promote the proportion of IL-1β-expressing microglia *in vitro* and *in vivo*, which contributes to the exacerbation of neuroinflammation (Reverchon et al., 2017, 2020). IL-33 administration also enhances LPS by primary microglia inducing abundant secretion of TNF-α and IL-6, but administration of IL-33 alone fails to stimulate microglia to release pro-inflammatory cytokines (Cao et al., 2018). Paradoxically, IL-33 administration suppresses IL-1β expression in microglial flows sorted from Plasmodium-infected brains with or without antimalarial drug treatment (Strangward et al., 2018). Similarly, antagonists of IL-33 exacerbate the EAE-induced release of TNF-α from microglia, which is subsequently inhibited by IL-33 supplementation (Xiao et al., 2018). There are at least three possibilities leading to these inconsistent findings. First, the dose of IL-33 treatment *in vitro* is variable (Reverchon et al., 2017; Cao et al., 2018); second, changes in microglial inflammatory cytokines have been assessed by different experimental techniques (Reverchon et al., 2017; Cao et al., 2018; Xiao et al., 2018); finally, other underlying factors may contribute to the opposing effects of IL-33 on microglial inflammatory responses in diverse CNS diseases but are not recognized (Cao et al., 2018; Strangward et al., 2018; Xiao et al., 2018).

Accumulating literature demonstrates an anti-inflammatory role for IL-33 in ischemic stroke (Korhonen et al., 2015; Yang et al., 2017; Jiang et al., 2018; Luo et al., 2018). Blockade of IL-33/ST2 signaling through IL-33 and ST2 gene deletions can lead to disruption of M2 polarization and promotes phenotypic changes to M1 microglia in the ischemic penumbra (Yang et al., 2017; Luo et al., 2018). Furthermore, besides decreasing the expression of M1 markers, exogenous IL-33 treatment increases M2 marker expression levels within peri-infarct microglia in brain sections (Korhonen et al., 2015; Yang et al., 2017; Luo et al., 2018). Apart from *in vivo* experiments, when primary microglia are exposed to oxygen and glucose deprivation, the IL-33/ST2 pathway significantly suppresses CD16 expression and slightly enhances CD206 expression in microglia (Jiang et al., 2018). Furthermore, ST2 deficiency induced downregulation of the transcription factor pSTAT6, which is considered an inducer of M2 microglia in microglia of the peri-infarct area (Cai et al., 2019; Xie et al., 2021). These results support that IL-33, whether endogenous or exogenous, enhances polarization toward M2 microglia in ischemic stroke. Despite different forms of IL-33 administration in diseases such as intracerebral hemorrhage, spinal cord injury, glioma, and AD, an increase in the percentage of microglia expressing M2 markers (Arginase-1 or CD206) is consistently induced by IL-33 treatment (Pomeshchik et al., 2015; Fu et al., 2016; Chen et al., 2019; De Boeck et al., 2020). In particular, IL-33 knockout contributes to a high degree of elevation of M1 resident microglia during the peak phase of EAE, but both exogenous recombinant IL-33 or IL-33 monoclonal antibodies have no effect on primary microglia stimulated brain homogenates from EAE mice (Xiao et al., 2018). Furthermore, IL-33/ST2 signaling and CD206-positive microglia are simultaneously induced by cannabinoids in carbon monoxide poisoning (Du et al., 2020).

Whether IL-33 leads to pro-inflammatory or anti-inflammatory cytokines secreted by microglia is debatable, but IL-33 has been found to be an essential regulator of M2 microglial polarization under multiple conditions. Thus, microglial activation state transitions induced by IL-33 provide a protective function in neuroinflammation except in gliomas, where M2 microglia are considered tumor-forming promoting immune cells (De Boeck et al., 2020). The regulatory mechanism by which IL-33 promotes the transition to the activated state of M2 microglia in the above diseases is still unknown.

## Conclusion

Although much of the literature mentions the close relationship between IL-33 and microglia, the systemic and precise effects of IL-33 on microglial function are unknown. Some studies have taken microglial activation and proliferation as indicators of disease severity assessment and have determined microglia in a non-specific manner, thus overlooking the dynamic transcriptome of microglia and macrophages and hiding the true interaction between IL-33 and microglial activation. On the other hand, convincing experiments support that IL-33 is a necessary and sufficient cytokine for microglial phagocytic processes and migratory activity; the dual role of IL-33 in microglial M1/M2 phenotypic changes and secretion of inflammation-related cytokines explains why IL-33 reactive microglia exhibit diverse inflammatory responses under different conditions or even in the same disease. One way to address this question is to elucidate the specific pathogenesis in these diseases and to precisely control IL-33 expression and dosage.

However, how IL-33 is involved in the functional expression of microglia remains poorly understood. By delving deeper into the molecular networks and transformations of cellular metabolism, a clearer picture of the regulatory role that IL-33 plays in the microglial functional state may be obtained.

## Author contributions

TX: writing—original draft preparation. XW and YW: writing—review and editing. YZ: writing—picture drawing. All authors contributed to the article and approved the submitted version.
